# Antioxidant potential of aqueous extract of *Phyllanthus amarus* in rats

**DOI:** 10.4103/0253-7613.51342

**Published:** 2009-04

**Authors:** R. Karuna, S. Sreenivasa Reddy, R. Baskar, D. Saralakumari

**Affiliations:** Department of Biochemistry, Sri Krishnadevaraya University, Anantapur - 515 003, Andhra Pradesh, India

**Keywords:** Antioxidants, lymphocytes, *Phyllanthus amarus*, single cell gel electrophoresis SCGE

## Abstract

**Objective:**

Increased levels of oxidative stress may be implicated in the etiology of many pathological conditions. Protective antioxidant action imparted by many plant extracts and plant products make them promising therapeutic drugs for free radical induced pathologies. In this study we assessed the antioxidant potential of *Phyllanthus amarus* (Euphorbiaceae).

**Materials and Methods:**

Experimental rats were divided into two groups: Control and *Phyllanthus amarus (P. amarus)* treated. Treated rats received *P. amarus* aqueous extract (PAAEt) at a dose of 200 mg/kg body wt/day for 8 weeks. After the treatment period of 8 weeks lipid peroxidation (LPO), vitamin C, uric acid and reduced glutathione (GSH) were estimated in plasma and antioxidant enzymes: Glutathione peroxidase (GPx), catalase (CAT) and superoxide dismutase (SOD) were also assayed. Genotoxicity of PAAEt was assessed by single cell gel electrophoresis (SCGE) of lymphocytes under both *in vitro* and *in vivo* conditions. The protective role of PAAEt against hydrogen peroxide (H_2_O_2_), streptozotocin (STZ) and nitric oxide generating system induced lymphocyte DNA damage was also assessed by SCGE.

**Results:**

PAAEt treated rats showed a significant decrease in plasma LPO and a significant increase in plasma vitamin C, uric acid, GSH levels and GPx, CAT and SOD activities. SCGE experiment reveals that PAAEt was devoid of genotoxicity and had a significant protective effect against H_2_O_2_, STZ and nitric oxide (NO) induced lymphocyte DNA damage.

**Conclusion:**

The results suggest the non-toxic nature of PAAEt and consumption of PAAEt can be linked to improved antioxidant status and reduction in the risk of oxidative stress.

## Introduction

Oxidative stress occurs in a cellular system when the production of reactive oxygen species (ROS) exceeds the antioxidant capacity of the system. Oxidative stress plays an important contributory role in the process of aging and pathogenesis of numerous diseases like diabetes, cancer, neurodegenerative diseases, and respiratory tract disorders.[1] Improved antioxidant status helps to minimize the oxidative damage, and thus can delay or decrease the risk for developing many chronic age related, free radical induced diseases.

*Phyllanthus amarus* (Euphorbiaceae) can be found in all tropical regions of the world including southern India and China. In many countries around the world plants in the genus *Phyllanthus* are used in folk remedies; therefore this genus is of great importance in traditional medicine. *P. amarus* has been claimed to be an excellent remedy for infective hepatitis.[2] It was reported to have anti-plasmodial,[3] antiviral,[4] anti-bacterial[5] and anti-diarrheal[6] properties. Extract of *P. amarus* exhibited protective action against CCl_4_ induced mitochondrial dysfunction[7] and hepatoprotective potential against ethanol,[8] alloxan[9] and cyclophosphamide-induced oxidative stress in rats.[10] *P. amarus* extract also possessed anti-tumor, anti-carcinogenic[11] and anti-inflammatory properties.[12] Methanol extract of *P. amarus* was reported to have hypoglycemic effect on alloxan induced diabetes mellitus.[9]

The generation and subsequent involvement of free radicals in a large number of diseases prompted us to study the antioxidant potential of PAAEt. In order to consider any substrate or plant extract as an effective antioxidant, it should act as antioxidant under both *in vivo* and *in vitro* conditions by decreasing the levels of oxidative damage to biomolecules, and it should render lymphocytes more resistant to oxidative challenges. Therefore, the present study was designed to explore the effect of PAAEt treatment on plasma antioxidant status and to evaluate the antigenotoxic potential of PAAEt against STZ, NO and H_2_O_2_ induced DNA damage in lymphocytes.

## Materials and Methods

### Chemicals

The chemicals used in the current study were procured from Sigma Chemical Co. (St. Louis, MO, USA), Koch-Light Laboratories (Huntingdon, Cambridgeshire, England), and SISCO Research Laboratories (Maharashtra, India). An aqueous extract of *P. amarus* (dry powder) was purchased from Chemiloids (manufacturers and exporters of herbal extracts), Vijayawada, Andhra Pradesh (India). Herb-to-product ratio is 10:1. The phytochemical screening of the PAAEt was carried out by the modified method of Das and Bhattacharjee.[13] The extract was dissolved in distilled water prior to use.

### Animals

Two- to three-month-old male albino Wistar rats weighing 130-150 gm, procured from Sri Venkateswara Enterprises (Bangalore, India), were acclimatized for 7 days to the animal house, and maintained at standard conditions of temperature and relative humidity, with a 12 h light/12 h dark cycle. Water and standard pellet diet were provided ad libitum. The current work was carried out after approval by our institutional animal ethical committee (Regd. no. 470/01/a/CPCSEA, dt. 24^th^ Aug 2001).

### Experimental design

In this experiment, a total of 16 rats were used. The rats were divided into two groups (control and *P. amarus* treated) of 8 animals each. *P. amarus* treated animals received ∼2 ml of PAAEt (200 mg/kg body weight) orally through gastric tube daily. The dose of PAAEt in the current study is based on preliminary experiments on anti-hyperglycemic effect in alloxan-induced diabetic rats. After 8 weeks of treatment, 1-2 ml of blood was collected from rats by means of a capillary tube through orbital sinus into heparinized Eppendorf tubes. Plasma was separated and used for biochemical analysis.

### Biochemical analysis

Glucose was estimated by glucose oxidase peroxidase method using the Span Diagnostic kit. Vitamin C was estimated by the method of Omaye *et al.*[14] and uric acid was estimated by enzymatic method using a Liquid GOLD kit. Reduced glutathione was estimated by Ellmans method.[15] The extent of lipid peroxidation was determined by the method of Utley *et al*.[16]

### Enzyme assays

Plasma GPx (EC 1.11.1.9), CAT (EC 1.11.1.6) and SOD (1.15.1.1) were assayed by following the methods of Rotruck *et al.,*[17] Beers *et al.*[18] and Soon and Tan[19] respectively. Protein was estimated by the method of Lowery *et al.*, using bovine serum albumin as standard.[20]

### Single cell gel electrophoresis

Experiments were performed on lymphocytes isolated from the blood of rats using histopaque-1077 technique, according to established methods.[21] After separation, lymphocytes were suspended in RPMI 1640 medium and counted over a hemocytometer and adjusted to nearly 5 × 10^5^ cells/ml of medium. The basic protocol for the comet assay was applied according to the modified method of Singh *et al*.[22] The modification includes a silver staining[23] of comets instead of ethidium bromide. The slides were coded for each sample and cells were screened on each slide under a standard transmission binocular microscope and DNA migration length was measured using an ocular micrometer.

### In vitro genotoxicity studies of PAAEt

Lymphocytes isolated from the blood of normal rats were incubated with PAAEt in RPMI medium (0.2, 0.4 and 0.6 mg/ml medium) at 37°C in CO_2_ humidified incubator for 1 h. After incubation the cells were centrifuged at 3000 rpm for 5 min to pellet the lymphocytes. The pellet was re-suspended in RPMI medium and the lymphocytes were subjected to SCGE.

### Effect of plant extract against in vitro oxidative stress caused by nitric oxide generating system/ STZ

The nitric oxide generating system consists of a freshly prepared mixture of 10 mM Tris-HCl buffer pH 7.4 prepared in saline and 30 mM sodium nitroprusside in 1.5:1 ratio. *In vitro* PAAEt pretreated (0.2 mg/ml RPMI medium) lymphocytes from normal rats were exposed to nitric oxide generating system (100 *μ*l/1 ml medium) or 10 *μ*l of freshly prepared STZ in PBS (to get a final concentration 80 *μ*g/ml medium) for 25 min and centrifuged at 3000 rpm for 5 min to pellet the lymphocytes and then the lymphocytes were subjected to SCGE.

### In vivo effect of PAAEt against H_2_O_2_ induced lymphocyte DNA damage

Lymphocytes separated from the blood of normal and PAAEt treated rats were processed for SCGE. DNA damage was induced *in vitro* by exposing lymphocytes to H_2_O_2_. Two slides were prepared for each sample, one was immersed in a solution of H_2_O_2_ in PBS (200 *μ*mol/L) for 5 min and the other was immersed in PBS alone. The slides were washed twice with PBS and then subjected to SCGE. The protective effects of the plant extract against H_2_O_2_, NO/STZ induced oxidative stress were screened by measuring comet tail length against controls.

### Statistical analysis

All the results are expressed as means ± SE. Statistical analysis of the data was performed by Student's t-test and *P* value < 0.05 was considered statistically significant.

## Results

### Phytochemicals

The phytochemical screening of PAAEt revealed the presence of flavonoids, phenolic acids, steroids/triterpinoids, alkaloids, tannins and anthocyanins.

### General observations

During the eight weeks of experimental period, control rats showed no significant change in blood glucose level (64.14 ± 1.8 mg/dl) with gradual increase in body weight (from 147 ± 2.5 to 232 ± 5.3 g. PAAEt treated rats also showed the same trend as control rats regarding plasma glucose (66.46 ± 4.36 mg/dl) and gain in body weight (from 150 ± 3.8 to 237 ± 3.6 g during experimental period. No visible side effects (respiratory distress, abnormal locomotion and catalepsy) were observed in PAAEt treated animals during the experimental period.

### Plasma antioxidants

The levels of plasma LPO, vitamin C, uric acid and GSH are presented in Table 1. A significant decrease (11.9%) in plasma LPO was observed in treated rats compared to control rats, whereas significant increase in plasma vitamin C (28.6%), uric acid (35.7%) and plasma GSH (41%) levels was observed in PAAEt treated rats compared to normal rats. The activities of plasma GPx, CAT and SOD of control and PAAEt treated rats are presented in Table 1. A significant enhancement in the activities of plasma GPx (13.4%), CAT (28.4%) and SOD (25.18%) was observed in PAAEt treated animals compared to control animals.

**Table 1:** Effect of *P. amarus* aqueous extract on plasma antioxidants

*Parameters n = 8*	*Control rats n = 8*	*PAAEt treated rats*
LPO (nmol of MDA formed/dl)	32.20 ± 0.35	28.36* ± 0.78
Vitamin C (mg/dl)	1.10 ± 0.03	1.43* ± 0.032
Uric acid (mg/dl)	4.65 ± 0.07	6.31* ± 0.055
GSH (mg/dl)	2.24 ± 0.05	3.16* ± 0.05
GPx (μg of GSH consumed/min/mg protein)	0.25 ± 0.01	0.29* ± 0.005
CAT (mmoles of H_2_O_2_ decomposed/min/mg protein)	0.54 ± 0.03	0.69** ± 0.04
SOD (U/min/mg protein)	53.61 ± 0.86	67.11** ± 2.5

Values are mean ± SE from 8 rats in each group;

* *P* < 0.05;

** *P* < 0.001

### DNA damage

*In vitro* exposure of lymphocytes at different doses of PAAEt did not result in comet formation in SCGE indicating non-genotoxic nature of PAAEt. Results presented in Table 2 indicate the effect of supplementation of PAAEt 8 weeks on peripheral lymphocyte DNA damage and their ability to resist exogenous H_2_O_2_ induced damage.

**Table 2:** Effect of *in vivo* treatment of *P. amarus* aqueous extract against H_2_O_2_ induced lymphocyte DNA damage

*Group*	*Control rat lymphocytes*		*PAAEt treated rat lymphocytes*	
		
	*Basal*	*H_2_O_2_ treatment*	*Basal*	*H_2_O_2_ treatment*
Percentage of cells showing migration	1.50 ± 0.17	57.75** ± 0.57	1.35 ± 0.18	8.25**^f^ ± 0.42
Tail length (μm)	0.89 ± 0.10	4.95** ± 0.09	0.75 ± 0.06	1.22*^f^ ± 0.07

Values are mean ± SE from 400 cells from 8 rats in each group; Values not sharing common superscript letter differ significantly from control group basal level

* *P* < 0.05;

** *P* < 0.001; A superscript letter

f indicates a significant difference (*P* < 0.0001) between H_2_O_2_ challenged control and PAAEt treated group

*In vivo* treatment of PAAEt has not resulted in lymphocyte DNA damage. A significant protection against H_2_O_2_ induced DNA damage was observed in the lymphocytes of PAAEt treated rats compared to normal rats (85.3% decrease in number of damaged cells and 75.4% decrease in tail length of comets).

*In vitro* antigenotoxic effects of PAAEt against STZ and NO induced DNA damage are presented in Table 3. PAAEt pretreated lymphocytes showed decrease in the number of damaged cells (89.2% and 91.1%) and length of comets (72.4% and 77.3%) against STZ and NO induced DNA damage respectively.

**Table 3:** Protective ability of *P. amarus* aqueous extract against STZ/NO induced lymphocyte DNA damage

*Group*	*Control lymphocytes*			*PAAEt pretreated lymphocytes*		
		
	*Basal*	*STZ*	*NO*	*Basal*	*STZ*	*NO*
Percentage of cells showing migration	0.50	97.50***	98.20***	0.50	0.5***^f^	8.25***^f^
	± 0.40	± 1.30	± 0.33	± 0.38	± 0.60	± 0.51
Tail length (μm)	0.85	4.28***	5.11***	0.85	1.18*^f^	1.16**^f^
	± 0.08	± 0.12	± 0.11	± 0.08	± 0.05	± 0.07

Values are mean ± SE from 400 cells from 8 rats in each group; Values not sharing common superscript letter differ significantly from control lymphocytes basal level

* *P* < 0.05;

** *P* < 0.01;

*** *P* < 0.001; A superscript letter

f indicates a significant difference (*P* < 0.0001) between STZ/NO challenged control lymphocytes an PAAEt treated lymphocytes

## Discussion

Non-toxic nature of PAAEt was evident with the unaltered trend of body weight and plasma glucose levels in PAAEt treated rats compared to controls. The antioxidant potential of PAAEt was revealed by a significant decrease in the plasma LPO and a significant increase in plasma non-enzymatic antioxidants, vitamin C, uric acid and GSH in PAAEt treated rats compared to control.

Recently, free radical induced LPO has gained much importance because of its involvement in several pathologies such as aging, atherosclerosis, diabetes, wound healing, liver disorder, inflammation etc. Protection of the cell membrane from LPO could prevent, cure or delay the aforesaid pathologies. The enhanced plasma GSH, vitamin C, uric acid levels of PAAEt treated rats may be responsible for the observed decrease in the extent of plasma LPO.

Glutathione, SOD and CAT protect the cell constituents from oxidative damage. Despite these extensive defense systems, biomolecule damage may still occur and persist within the cell. The significant increase in the activities of SOD and CAT suggests a greater level of endogenous antioxidant associated with the PAAEt treatment resulting in an enhanced free radical scavenging activity. Plants are the sources for a wide variety of compounds like flavonoids and polyphenols. These compounds may be responsible for increasing antioxidant status.

The first screening of any compound, drug or potential nutraceutical starts with the genotoxicity test.[24] Endogenous levels of DNA damage remained unchanged under *in vivo* and *in vitro* treatment of PAAEt revealing that PAAEt is devoid of genotoxic and pro-oxidant property. The decreased levels of H_2_O_2_ induced DNA damage in PAAEt treated rat lymphocytes is attributed to the increased scavenging of H_2_O_2_ derived ROS by enhanced antioxidants.

Recent studies suggested that ROS, including superoxide (O_2_°^−^), H_2_O_2_, hydroxyl radical (°OH) and °NO play a central role in the mechanism of DNA damage and cytotoxicity of STZ.[25] Nitric oxide, generated from sodium nitroprusside in aqueous solution at physiological pH, interacts with oxygen to produce nitrite ions.

The decreased levels of STZ/NO induced DNA damage in PAAEt pretreated lymphocytes (*in vitro*) may be attributed to the possible direct antioxidant capacity of PAAEt. Phytochemical constituents of *P. amarus* may be responsible for scavenging ROS and protecting the DNA from ROS induced DNA damage. The concept of synergy is central to the holistic approach. The popular modern concept trend to isolate pure compounds may not achieve the desired results as observed in the natural version. Once an active principle is isolated from the natural product without its synergical colleagues to support and/or balance its action, it may lose its character as present in its natural form.

## Conclusion

Our present study indicates that PAAEt is devoid of genotoxicity and pro-oxidant property. The enhanced antioxidant status observed in PAAEt treated rats and its protective role against H_2_O_2_, STZ and nitric oxide generating system induced DNA damages might be due to the effect of different types of active principles acting individually or synergistically, each with a single or a diverse range of biological activities against oxidative stress, a widely recognized factor in many degenerative diseases.
